# Quality Control of Jinhua Ham from the Influence between Proteases Activities and Processing Parameters: A Review

**DOI:** 10.3390/foods12071454

**Published:** 2023-03-29

**Authors:** Shiqi Hu, Xinglian Xu, Wangang Zhang, Chunbao Li, Guanghong Zhou

**Affiliations:** 1 Key Laboratory of Meat Processing and Quality Control, Ministry of Education, Nanjing 210095, China; 2 Key Laboratory of Meat Processing, Ministry of Agriculture and Rural Affairs, Nanjing 210095, China; 3 Jiangsu Collaborative Innovation Center of Meat Production, Processing and Quality Control, College of Food Science and Technology, Nanjing Agricultural University, Nanjing 210095, China

**Keywords:** endogenous proteases, dry-cured ham, process parameters, protein degradation, flavor

## Abstract

Endogenous proteases are significant for Jinhua ham quality. Protein degradation affects the chemical traits, texture and the formation of flavor substances. Protease activities are affected by different process parameters, such as processing temperature, maturation time, salt content and the drying rate. They affect ham quality, which can be controlled by process parameters. The influences of key factors on Jinhua ham quality are briefly summarized, which can provide a theoretical basis for the selection of specific parameters in dry-cured ham processing. Furthermore, some suggestions are proposed for correcting and improving the flavor and textural defects of ham, yet the effectiveness depends on the operating conditions. The determination of enzyme activity is not real-time and unsupervised at the moment. Future research will focus on the determination of the actual endogenous protease activity and the quantitative relationship between the enzyme activity and main processing parameters.

## 1. Introduction

There are various types of dry-cured ham, and the ripening process is critical for flavor development. Among them, Jinhua ham is an important part of traditional Chinese meat products with a long history. Likewise, Parma ham from Italy, Iberian ham, and Serrano ham from Spain are also globally recognized. The unique production process for each type of dry-cured ham contributes to their characteristic color, texture and flavor [1]. In the modern procedure of Jinhua ham, salting is carried out by adding 6 g of NaCl per kilogram of raw ham. This is followed by processes such as washing, air-drying, ripening, and post-ripening [2]. It requires a few months, while the processing time for ham from Italy and Spain is even longer. For instance, Iberian dry-cured ham needs 18–24 months for manufacturing. Furthermore, compared to Parma ham and Iberian ham, the salt content is high in the finished products of Jinhua ham. It reaches 12%, while Parma ham is 4.2–6.2% and Iberian ham is around 6.5% [3,4]. It is worth noting that proteolysis is critical to the formation of ham flavor and texture, as demonstrated by numerous studies. This process is primarily induced by muscle endogenous proteases [5,6].

Protease activities are affected by a variety of factors, and the changes in either intrinsic or extrinsic factors have a probable effect on endogenous protease activity [7]. Zhao et al. [8] recorded the changes of cathepsin B and L activities during Jinhua ham processing, and they revealed that temperature, pH value and salt content had significant effects on them. Zhou et al. [9] found that sodium chloride had a significant inhibitory effect on cathepsins activities, and Jinhua ham with reduced salt exhibited higher sensory scores. Moreover, the literature from Zhou et al. [10] showed intenser taste and richer attributes in modern-processed Jinhua ham, and the activity of endogenous protease was found to vary with changes in ambient temperature and relative humidity. Arnau et al. [11] studied the addition of lipases and proteinases during the process of fermented sausages. Based on the above description, it can be found that a comprehensive understanding of the influence between proteases activities and processing parameters is necessary to optimize Jinhua ham processing [12,13]. Internal factors affecting protease activity in ham include pH level, weight, and fat content, while extrinsic factors are composed of processing temperature, maturation time, salt content, and the use of new equipment. The changes in these factors are of great practical significance in predicting and regulating the activity of endogenous protease [14,15].

The processing technology and competitiveness of Jinhua ham are considered to be inadequate when compared to Italian and Spanish hams. Currently, there is a significant amount of literature exploring salt reduction, technological modernization and the time saved in the production [16,17]. It is important to note that the activities of muscle endogenous proteases are influenced by multiple factors. However, there is a lack of information available regarding how to improve ham quality through the influence between protease activity and processing parameters. This paper summarizes the contribution of endopeptidases and exopeptidases to Jinhua ham flavor and texture, and discusses how processing parameters affect protease activity. The aim of this study was to explore the relationship between endogenous protease activity, processing parameters, and ham quality. The ultimate goal was to identify the factors that influence protease activity and optimize processing conditions to achieve higher quality products.

The rest of this paper is structured as follows: Section 2 provides information about the different endogenous proteases in Jinhua ham, including calpains, cathepsins, dipeptidases, tripeptidases and aminopeptidases. In Section 3, the contribution of endogenous proteases to the texture and flavor of Jinhua ham is clarified. Additionally, Section 4 shows the association between enzyme activity and ham processing parameters. Various influencing factors are involved, such as raw material, processing temperature, NaCl content, processing time and the application of non-destructive techniques. Finally, the conclusion of this paper is presented in Section 5.

## 2. Compositions and Characteristics of Proteases

**Figure 1:**
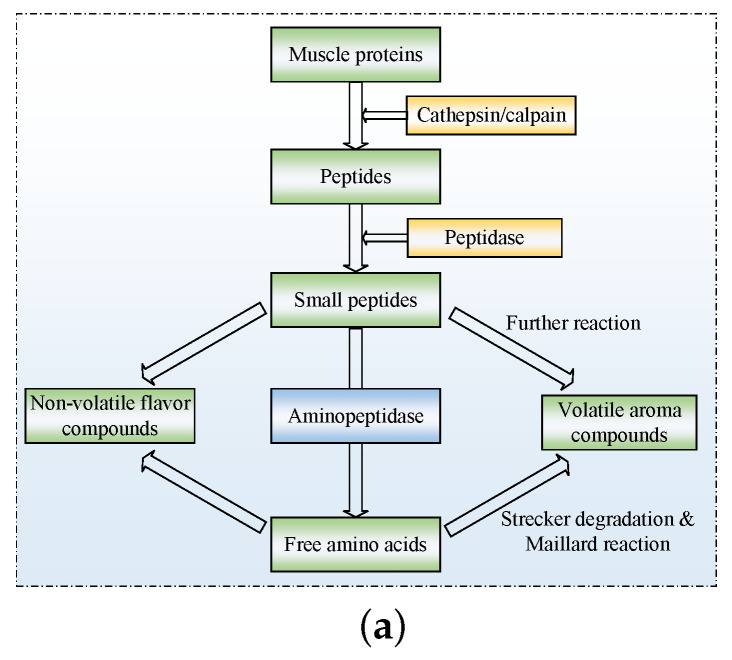

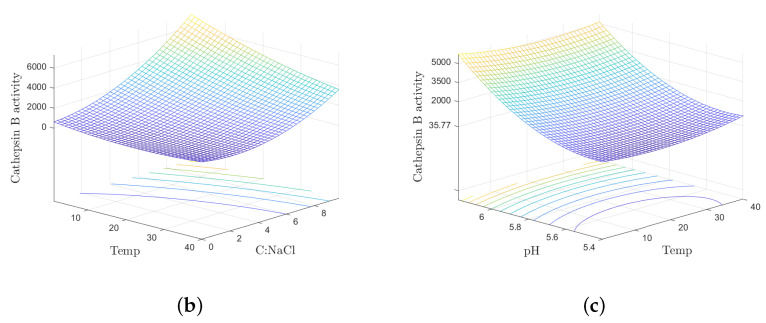
The formation of flavor substances in Jinhua ham and the interactive effect between processing parameters. (**a**) Formation of flavor substances in Jinhua ham and the role of endogenous protease [18]. (**b**) Effects of temperature and pH value on cathepsin B activity. (**c**) Effects of temperature and salt content on cathepsin B activity [8].

The section provides an overview of different endogenous proteases in Jinhua ham muscle. Figure 1a shows their role in the degradation process of muscle protein, explaining the formation of flavor substances. Firstly, the skeleton proteins are degraded by calpains and cathepsins. Then, the peptides produced above are degraded into small molecule peptides under the further action of dipeptidases and tripeptidases. Ultimately, they are degraded into free amino acids by aminopeptidases and carboxypeptidases.

### 2.1. Calpains

**Figure 2:**
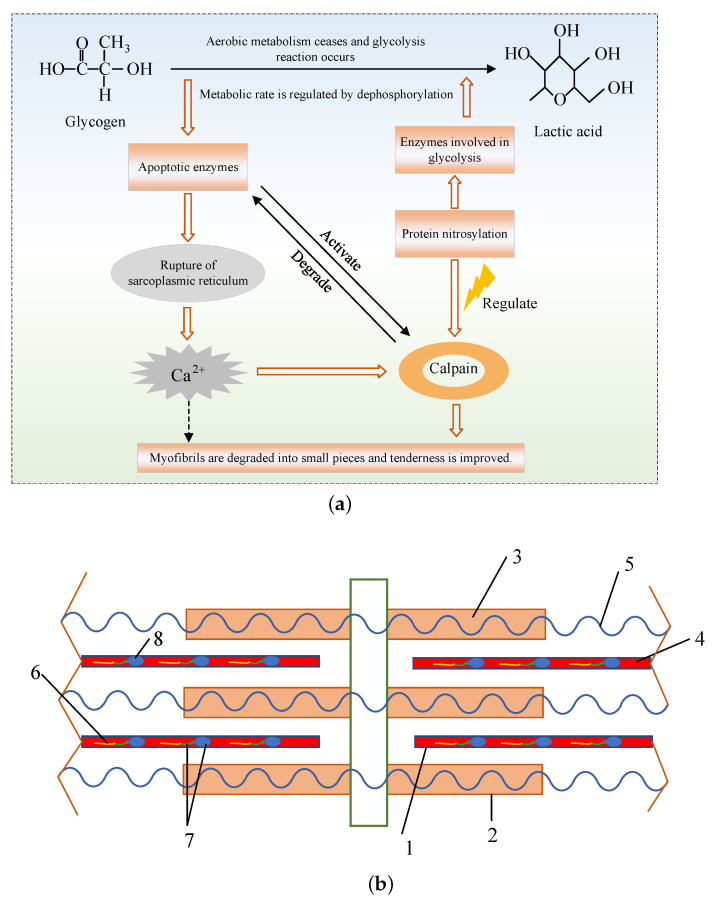
The role of calpains in meat tenderization and their effect on the structure of myofibrils [23]. (**a**) The mechanism of meat maturation involving calpains. (**b**) The structure of myofibrils (myofibrillar protein related to postmortem maturation are marked separately: 1-thick myofilament; 2-thin myofilament; 3-myosin; 4-Actin; 5-Titin; 6-Tropomyosin; 7-Troponin complex; 8-Troponin.

Calpain is a major contributor to meat tenderness improvement. It is a kind of protease and it can degrade various muscle proteins, mainly myofibrillar proteins. Numerous studies have found that calpain improved the tenderness of meat by degrading skeletal proteins in muscle cells such as desmin, fibrin, troponin-T and connexin. Therefore, the muscle fibers are eventually degraded and the shearing force of the skeletal muscle reduces, resulting in changes in the structure of myofibrils and the improvement of meat tenderness [19]. Calpain is a calcium ion-dependent protease and its activity can be activated by calcium ions. It can be divided into μ-calpain (micromolar level) and *m*-calpain (millimolar level) according to the calcium ion concentration required to activate the proteins. Calpain has been widely studied in the post-mortem maturation of meat. The role of calpain in the tenderization and the myofibrillar protein related to meat maturity after slaughter are shown in the Figure 2. It is extremely sensitive to temperature and pH, and the optimum temperature is about $25{\;}^{\circ }C$. Due to some existing factors, such as the autolysis and poor stability of calpain, and the pH condition, most of its activity is completely lost after the long maturation [20,21]. Nevertheless, what cannot be ignored is that calpains can greatly promote the degradation of the skeleton protein and the destruction of the integrity of myofibril structure, which is conducive to the further action of other proteases [22].

### 2.2. Cathepsins

Cathepsins are considered to play a major role in the protein degradation during the processing of Jinhua ham, especially cathepsin B and L. Unlike calpain, which plays a crucial role in the maturation and tenderization and the activity of which is almost completely lost at the end of ham’s elaboration procedure, cathepsin keeps active throughout the whole processing of dry-cured ham. At present, nearly 20 cathepsins have been isolated and identified. Some researchers have evaluated the activities of cathepsins and protein degradation as well as the enzymatic properties in hams. Partial characteristics of the common lysosome cathepsins existing in animal muscles are listed in Table 1. Venugopal et al. [24] examined the optimal pH and temperature, molecular weight and the isoelectric point of cathepsin D. Zhou et al. [25] discussed the kinetics of enzyme-catalyzed reactions of cathepsin L. Moreover, myosin, troponin, tropomyosin and actin can be degraded by cathepsin B. Myosin heavy chain, α-actin, actin, and the T subunit of troponin will be degraded by cathepsin L [26].

Cathepsin B has been extracted and purified from the muscle, liver and pancreas of carp, bovine spleen, pig liver, mouse liver, human liver, salmon, tilapia, silver carp, and other fish muscles. Cathepsin B is composed of two molecular structures—namely cathepsin B1 and cathepsin B2—and the optimum temperature is about $40{\;}^{\circ }C$. In animals, cathepsin B usually exists in the following three forms: an inactive enzyme-endogenous inhibitor form, an inactive or low-activity precursor form, and the active mature monomer form. Cathepsin L has several complex structures. Iodoacetic acid, leupeptin, and anti-protease have a strong inhibitory effect on its activity, but aprotinin and sulfonyltoluene fluoride have no inhibitory effect [28]. It is excessively unstable at neutral and alkaline pH and easily denatured and inactivated. The optimum temperature of cathepsin L is 40–45 ${}^{\circ }C$, and the high activity occurs at 20–60 ${}^{\circ }C$. Its activity decrease rapidly at $70{\;}^{\circ }C$ and cathepsin L is completely inactivated at about $80{\;}^{\circ }C$. Cathepsin B and L have extensive effects on protein degradation. For example, actin can be degraded into peptides released from the N-end and C-end of the protein by cathepsin B [29].

Cathepsin H possesses endonuclease and exonuclease properties with a molecular weight of 26–28 kDa. It has maximum vitality at pH 7.0. Kaur et al. [30] confirmed that cathepsin H existed in animal skeletal muscle. In addition, for degrading muscle proteins and generating taste substances, cathepsin H is critical in Jinhua ham [31]. Cathepsin D is less stable than cathepsin B and L. Sárraga et al. [32] found that its activity decreased quickly during the production of dry-cured ham, which showed that the residual activity reached 10–20% of the raw hams after processing for 2.5 months. Cathepsin D may only be active during the first few months of processing.

### 2.3. Tripeptidases and Dipeptidases

In dry-cured ham, small peptides in the muscle are produced by peptidyl peptidases, including tripeptidylpeptidase (TPP) and dipeptidylpeptidase (DPP). TPP is an enzyme that can hydrolyze different tripeptides from the amino terminus of proteins and polypeptides. TPP I is 46kD glycoprotein and exists in the lysosome organelles with an optimal pH of 4.0. Substrate Gly-Pro-X can be hydrolyzed, where X is any kind of amino acid and hydrophobic amino acids are preferred [33]. In addition, TPP I is considered to be an aspartic peptidase, and its sequence has a certain similarity to carboxypeptidase belonging to the peptidase family. TPP II is an oligomer composed of 138kD subunits and the optimum pH is 6.5–7.5. TPP II can hydrolyze peptides to produce a variety of tripeptides, and Ala-Ala-Phe is a typical tripeptide preferentially hydrolyzed by TPP II [9].

DPP is always highly active throughout ham processing because of its diverse types, wide range of action and good stability [34]. It is the protease that generates dipeptide products from the amino terminal under the hydrolysis of peptides and proteins. DPP can be divided into four different types: DPP I, DPP II, DPP III and DPP IV. In terms of distribution, DPP I and DPP II are found in the lysosome. DPP III is present in the cytoplasm, and DPP IV is located on the cell membrane. Among all dipeptidases, DPP I exhibits high activity and is the most active enzyme. DPP I has good stability and plays an important role in the whole processing of dry-cured hams [35]. Wang et al. [36] have obtained a higher degree of protein degradation employing the raw material with higher DPP I activity, which is related to a higher amount of peptide production. It is consistent with a fact that DPP 1 activity is needed in the flavor formation process. DPP II plays a certain role, mainly before the period of ripening rather than throughout the whole ham processing [37]. DPP III reaches its maximum activity at a pH of 8.0 and a temperature of $40{\;}^{\circ }C$. Its activity decreases rapidly under acidic conditions and decreases significantly when the optimum temperature is exceeded [38]. DPP IV is a dipeptidase that is second only to DPP I in activity and it has a positive effect on the proteolysis.

### 2.4. Aminopeptidases

Free amino acids are due to the action of aminopeptidases, which are crucial in the formation of ham taste. Aminopeptidases cannot be separated from the proteolytic action when talking about ham ripening. Aminopeptidases are mainly involved in the later stages of dry-cured hams, which usually exist in the cytoplasm with complex structures and various types. Five main types are closely related to the processing of Jinhua ham and have been studied—namely alanyl amino peptidase (AAP), arginyl amino peptidase (RAP), leucyl amino peptidase (LAP), tyrosine acylaminopeptidase (TAP) and pyroglutamylaminopeptidase (p GAP) [39]. All of them show high activity between 30 ${}^{\circ }C$ and 40 ${}^{\circ }C$ and pH 5.0–7.0, which is similar to the ripening conditions of Jinhua ham. The analysis of potential activities of various aminopeptidases showed that AAP, RAP and LAP are active and maintain high residual vitality [18]. In the dry-cured hams, they are the main reasons for the accumulation of free amino acids. The actual effects of the three aminopeptidases are also different due to their substrate specificity.

AAP is the most abundant and important aminopeptidase in skeletal muscle sarcoplasm, accounting for 80–83% of the total activity. It has broad substrate specificity for aromatic amide bonds, aliphatic amide bonds and basic amide bonds. In comparison, the substrate range of LAP and RAP is much narrower. RAP is specific for basic amide bonds and it mainly hydrolyzes peptides with alkaline amino acids ends such as arginine and lysine [40]. The activity of LAP is very stable and high activity is still displayed in the later stage of dry-cured ham processing, so it plays a leading role in the formation of free amino acids. The concentration of free amino acids in the muscles continues to increase during the whole processing, especially for glutamic acid, arginine, alanine, aspartic acid, leucine, valine and lysine. The aminopeptidases activities of AAP, RAP and LAP are closely related to this increase in the Jinhua hams.

## 3. Action of Proteases for Improved Ham Quality

### 3.1. Protein Degradation

Intense proteolysis during the processing of Jinhua ham had been clarified [41,42]. Zhao et al. [26] determined the content of total nitrogen, total soluble nitrogen, non-protein nitrogen and soluble protein nitrogen for analyzing muscle protein degradation. The results showed that the soluble proteins are mainly degraded in the complex hydrolysis process, and are degraded faster than insoluble proteins. Sforza et al. [43] suggested that proteolytic enzymes lead to an accumulation of low molecular weight compounds. Zhao et al. [44] also indicated that the total concentration of free amino acids increased significantly during the storage of Jinhua ham, and the decisive role of aminopeptidases in the production of free amino acids was confirmed.

Yang et al. [45] found that higher moisture content promoted protein hydrolysis and the pH increased slightly to the range of 6.3–6.5, which was suitable for the action of many endogenous proteases in the muscle. Proteolysis index (PI) expresses the degree of proteolysis. Zhou et al. [46] ascertained that the diverse processing materials and processing technologies were important reasons for the difference in PI values. The significantly higher PI value is found in the defective ham, indicating more intense protein degradation. Simultaneously, cathepsin B and B + L with higher residual activity were detected in defective ham. Cathepsins led to stronger degradation in sarcoplasmic proteins and myofibril proteins.

### 3.2. Peptide and Free Amino Acid Generation

Small molecular peptides and free amino acids contribute to the ham flavor. Flavor is one of the most important quality indicators of meat products, and it is also a significant factor in attracting consumers. In the last 20 years, plenty of work has been carried out on the formation mechanism of dry-cured ham flavor. The results indicate that proteolysis and lipolysis are the main biochemical reactions in the generation of flavor or flavor precursors in ham processing. Proteolysis is dominated by endogenous proteases in view of the low microbial counts found inside hams [47]. The key part of dry-cured ham taste consists of small molecule peptides and free amino acids. Umami, sweetness, sourness and bitterness properties depend on their content and proportion. Figure 1a shows that the resulting taste-related peptides and free amino acids provide a taste for hams.

**Figure 3:**
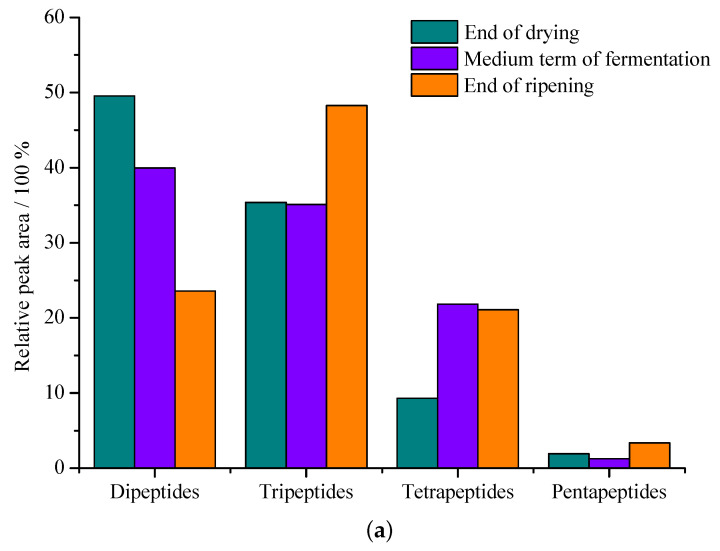

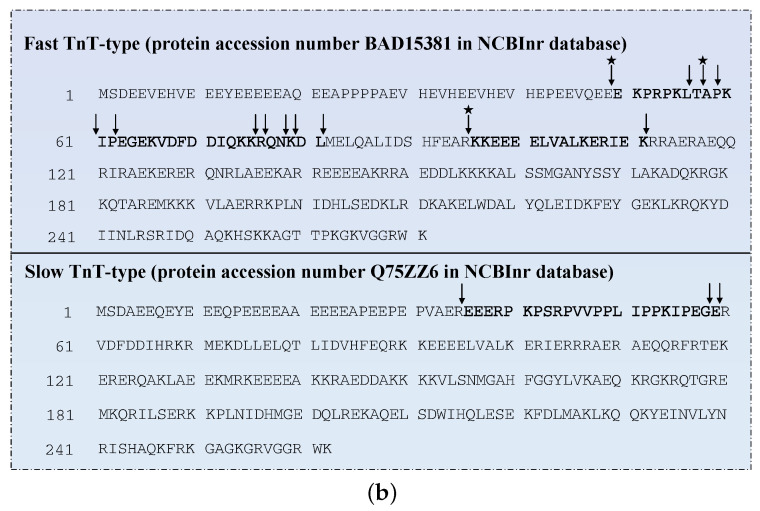
The change trend of small peptides in Jinhua ham and the breaking sites of enzymes. (**a**) Comparison of changes of main small peptides relative area peaks from the three periods of Jinhua ham [48]. (**b**) Primary sequence of fast and slow troponin T proteins from porcine skeletal muscle. Letters in bold indicate the position of the identified peptides in the protein. Black arrows indicate endo and exo cleavage sites of the peptides identified in this study. Arrows marked with an asterisk indicate agreement with calpains cleavage site reported [51].

In many investigations, free amino acids, polypeptides and oligopeptides provide a unique flavor and quality in dry-cured hams, and they originate from extensive proteolysis of endogenous peptidases. A large amount of peptides increase after maturation and the change trend of small peptides in Jinhua ham is shown in Figure 3a. The accumulation of peptides derived from myosin is similar to the change in the total peptide content number, and the content of small peptides increases significantly, especially in the latter stages of ripening and post-ripening. Xing et al. [48] extracted and identified 213 peptides from Jinhua ham, and showed that polypeptide substances are mainly generated from myosin, troponin and actin. Huan et al. [49] surveyed the processing of Jinhua ham and found that 43, 46 and 63 major small peptides were obtained at different processing stages with molecular weights ranging from 204.1 to 1774.0 Da.

Recently, the use of mass spectrometry (MS) and peptomics methods has been able to better identify the source of the protein and the degradation site of the peptide. Mora et al. [50] identified the peptide sequences using LC-MS/MS. The dipeptides of DR, EN, HP, PL, IM, IQ, VF and the tripeptides of ALN, DPN, GHP, HGG, KLR, VGS were produced by creatine kinase degradation. The protein fragments of AQ, II, RG and IIP, RGA could originate from glyceraldehyde 3-Phosphate [51]. In addition, analysis of the correlation between the breaking sites of small peptides and enzymes in ham muscle provide better knowledge of the role of exopeptidase, which is shown in Figure 3b.

Furthermore, the relationship between various small peptides and the taste characteristics of ham is evident, and mainly depends on the length of the peptide chain itself, the composition and sequence of amino acids, and the original taste of their constituent amino acids [52]. The sensory properties of small peptides (generally peptides with molecular weight less than 3KDa) contain sweetness, sourness, saltiness, umami and bitterness. Aristoy et al. [53] focused on the taste characteristics of the peptides with a molecular weight less than 3 KDa, and they pointed out that the peptides with a molecular weight of about 1.8 kD exhibited a bitter taste. However, the molecular weight between 1.5 and 1.7 kD has an umami taste, and lower peptides (below 1kD) possess a slightly sour taste. By the action of muscle dipeptidases especially DPP I and DPP II [54], some di-peptides such as Ala–Gln, Arg–Gly, Asn–Pro, lle–Leu, Ala–Gly, Ser–Gly and Ser–Gln are released from the N-terminal probably. In addition, tripeptides such as Ile–Ile–Pro, Arg–Gly–Ala, Gly–Asn–Pro, Gly–Ala–Gly and Gly–Pro–Gly are detected and released through the action of TPP I.

### 3.3. The Microstructure of Muscle

Not only protein composition, but also the structure, is affected by the action of proteolytic enzymes. It is noteworthy that protein degradation leads to the production of protein polymers or fragments [55]. Wu et al. [56] examined the changes in protein structure during the heating process of meat products. Hydrogen bonds, hydrophobicity and disulfide bonds are the forces for stabilizing the protein structure. Sun et al. [57] identified the structural changes and discussed the effects of protein oxidation on the myofibril protein emulsification. It is proved that the addition of oxidants enhanced the surface hydrophobicity of myofibrillar protein. Therefore, the investigation into microstructure in ham muscle tissue is as important as postmortem storage, which provides valuable information for ham manufacture. In previous research, we performed SDS-PAGE electrophoresis experiments on the sarcoplasmic and myofibril proteins extracted from Jinhua ham. The analysis showed that the electrophoresis band of sarcoplasmic and myofibrillar proteins decreased sharply, which was due to the important role of cathepsin B and L in the processing of Jinhua ham.

**Figure 4:**
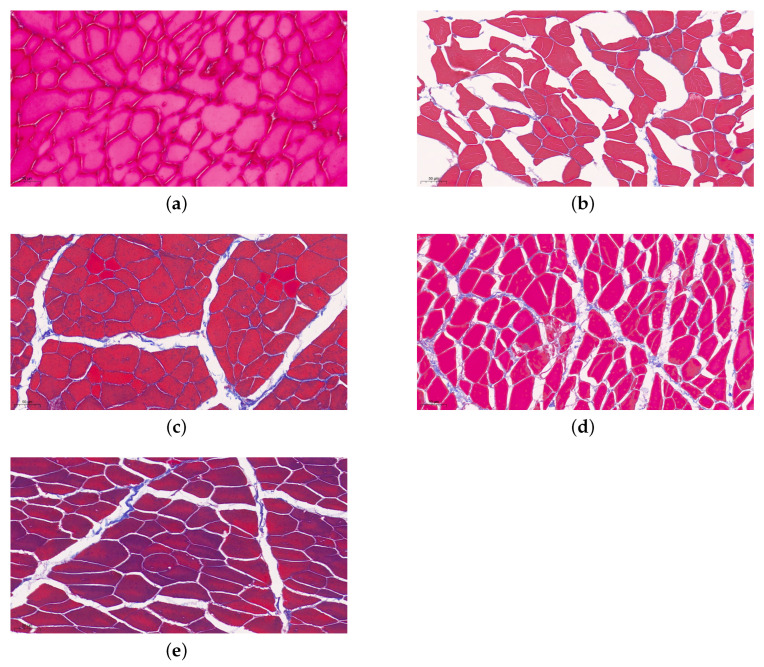
Masson staining results show the changes of muscle fibers in Jinhua ham from raw ham to finished stage (20×). (**a**–**e**) Identical letters represent the period of raw ham, post-salting stage, the medium term of ripening, the latter stage of ripening and the one-year-old hams respectively [59].

Furthermore, Harkouss et al. [58] determined the texture characteristics of dry-cured ham and highlighted the effect of endogenous proteolytic enzymes on muscle ultrastructure by electron microscopy. The model and relationship between structural parameters and proteolysis were investigated, which can be applied to any processing stages of Jinhua ham. As shown in Figure 4, the muscle fibers during different processing periods change gradually in the histological characteristics. In the beginning, the cross-section of the ham muscle tissue is relatively dense and the tissue structure is complete. The internal muscle fibers are thin and tightly arranged, which indicates that the higher the density of muscle fiber, the better the water retention and the finer the meat quality. After salting, moisture loss is accelerated with time, leading to muscle fibers atrophy and large gaps. On the contrary, the cross-section of ham muscle tissue tends to loosen and muscle fibers become thicker. Moreover, the hams, which have an excessive proteolysis due to marked protease activity, are prone to showing a softer tissue structure. In these mushy samples, the integrity of muscle cells and connective tissues disappears and the typical cellular structure can not be easily observed.

### 3.4. Endogenous Proteases and Texture Development

Texture defects of dry-cured ham induced by endogenous proteases are highlighted, such as pastiness, adhesiveness and softness. The myofibrillar proteins are destroyed through proteolysis, which constitutes the texture of dry-cured ham. Generally speaking, the texture property of Jinhua ham is a comprehensive impression that is mainly comprised of hardness, brittleness, adhesiveness, elasticity and cohesion. In Jinhua ham, the hardness, adhesiveness and chewiness increase significantly (p<0.05), yet there is no significant difference in elasticity. It suggested that the difference in texture is related to the water content, salt content and the degree of protein hydrolysis [60]. What is more, excessive proteolytic activity may create damage in the structure, leading to pastiness and adhesiveness. Pastiness is an oral sensation described as the feeling of flour and water paste during mastication. This is the major texture problem in dry-cured ham with a 10% incidence in the industrial production. Hernández-Ramos et al. [61] displayed that the proteolytic index was the best parameter for characterizing the relationship between processing conditions and texture of dry-cured ham. Another reason for softness is incomplete balance of moisture inside the muscle and the insufficient drying, which results in excessive moisture. Zhou et al. [12] showed that the adhesiveness was negatively correlated with the moisture content, and reducing the moisture content was conductive to improving the adhesiveness of dry-cured ham. In contrast, stiffness means that the structure of dry-cured ham is too hard, which results from the excessive drying of the dry-cured ham processing. High-pressure processing (HPP) treatment has been confirmed to decrease both pastiness and viscosity values [43]. After rheological analysis, Coll-Brasas et al. [62] proposed an in-vitro approach, which allowed faster, reproducible, and less expensive measurements for the pastiness defect in dry-cured ham. Contreras et al. [63] examined the use of ultrasound to assess pastiness and determined dry-cured ham non-destructively. This technology is effective at quickly detecting and separating defective hams to ensure product homogeneity and high quality.

### 3.5. Endogenous Proteases and Flavor Development

**Figure 5:**
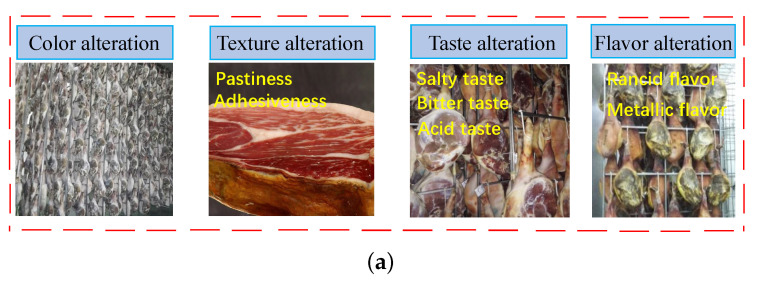

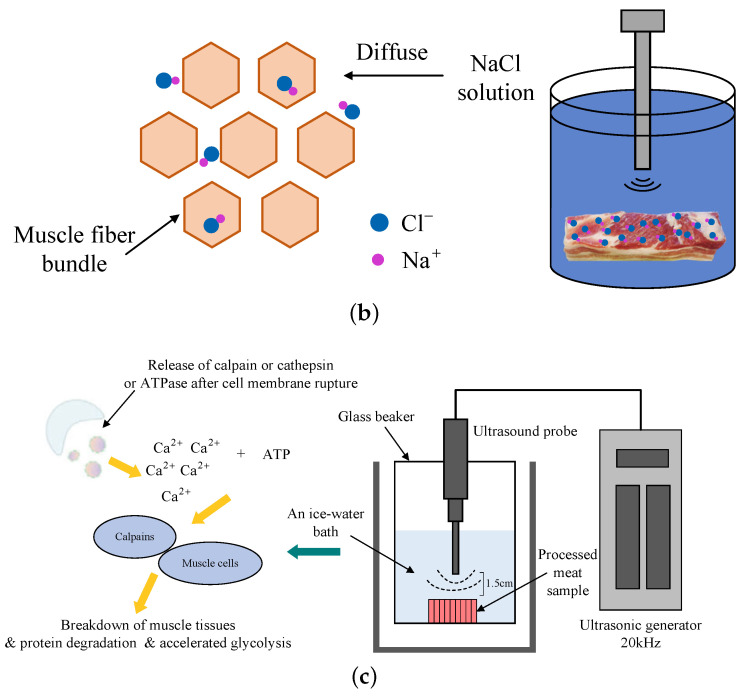
The possible sensory defects in Jinhua ham, application and principle of ultrasound in meat products processing. (**a**) The possible sensory defects in Jinhua ham [66]. (**b**) Schematic diagram of NaCl diffusion during the salting processing in the muscle promoted by ultrasound wave. (**c**) The processing of the release and activation of proteases promoted by ultrasound [67,68].

Strong endogenous enzymatic action is often a key factor in the development of sensory defects. In the time-consuming production of Jinhua ham, the degradation of muscle protein and degradation products will affect the texture, aroma and taste of the ham directly, which is dominated by the role of endogenous proteases [64]. The common defects in the ham quality are shown in Figure 5a. First of all, excessive saltness in the finished products is an urgent problem for the development of ham manufacturing. The defect in the ham quality is excessive salt, which is caused by the high salt content in the finished products. Meanwhile, NaCl is a potent inhibitor of most proteases involved in ham muscle and higher salt concentration suppresses putrid microorganisms reproduction inside and on the surface. However, the incidence of excessively soft and mushy hams will increase when sodium chloride is reduced, and the shelf life and safety of products is also not as expected [65]. Therefore, it is crucial to coordinate the endogenous protease activity and the salt amount.

Besides excessive saltiness, bitterness and sourness in Jinhua ham have usually produced an undesirable taste experience, and they generally appear with higher levels of proteolysis. The bitter hydrophobic free amino acids and short peptides accumulate massively in the highly proteolyzed hams. In an early study by Virgili et al. [69], high bitterness perception in aged ham was accompanied by pronounced endopeptidase activity, which was associated with high levels of methionine, asparagine, and isoleucine. This is in agreement with other research [70,71], so the ham can be effectively prevented from having a bitter taste by controlling the proteolytic index.

Additionally, dry-cured ham has also had an insufficient or even poor flavor. This is caused by an insufficient processing time to complete the necessary biochemical reactions, especially protein and lipid degradation reactions. As a result, the accumulation of flavor precursors and flavor substances in the muscle is scarce. In order to get round the quality defect of insufficient flavor, it is necessary to ensure a longer period for maturation (at least three months). Therefore, the selection of processing parameters is intricate and comprehensive. The biochemical changes that occur in the muscle tissues are quite intricate, so it is necessary to implement a complete and systematic control of the entire processing parameters to prevent the quality defects.

## 4. Key Parameters Influencing the Activities of Muscle Endogenous Proteases

### 4.1. Raw Material

Raw materials lead to different enzyme activities, including the breed, age, physical and chemical properties of the muscles. GarcÍa-Garrido et al. [72] showed that the reasons leading to different enzyme activity in raw materials are breed, age, physical and chemical properties of muscles. In several different ham types, Parma ham is made from Large White, Landrace and Duroc. Typical Spanish Iberian ham is mainly taken from Pure Iberian and Duroc males, and China Jinhua ham is made from “Liangtou Wu” pigs. The quality of the raw materials affects the rate and extent of biochemical reactions. Muscle properties, including pH, water content, salt concentration and tissue composition, are important factors affecting enzymatic activity. The biceps femoris (BF) and semimembranosus (SM) are two important muscle types with which to track the characteristics of dry-cured ham. The former is located inside and it is covered with a thick layer of subcutaneous fat one side, which slows the permeability of sodium chloride. The slow increase of salt in BF muscles throughout the process contributes to higher proteolytic activity [58]. On the contrary, SM muscles are close to the surface without fat coverage, resulting in fast absorption during salting. These two muscles have different protease activities, as well as intensive proteolysis [73]. Moreover, pig sex is also a factor in salt absorption and processing losses in dry-cured ham. The castration helps to improve the sensory quality of dry-cured ham [74].

PH value has an influence on the rate of an enzyme-catalyzed reaction directly and it plays a crucial role in ham manufacture. The pH value of raw material recommended for ham manufacture are between 5.6 and 6.2. With regard to the influence on enzymes, a low pH in hams is more prone to releasing cathepsins from lysosomes into muscle and enhances the activity of certain enzymes [75]. High pH values accelerate the growth and reproduction of microorganisms, which are also not suitable to be regarded as raw materials for Jinhua ham manufacture. For example, Guerrero et al. [76] discovered that the pastiness occurrence is associated with PSE meat. In response to the problem of softness in the dry-cured ham, the activity of cathepsin in fresh hind legs demand to be checked. Specifically, for legs with overhigh cathepsin activity, the amount of sodium chloride should be increased to inhibit protein degradation.

### 4.2. Processing Temperature

The temperature during the processing of Jinhua ham affects the enzyme activity directly, and has been listed in Table 2. Some researchers have found that the protease is the most active in the optimum temperature range with the pH, salinity and other conditions unchanged. For example, Moin et al. [77] reported that the optimum temperature of cathepsin B was about $40{\;}^{\circ }C$. Hu et al. [59] detected that cathepsins activities varied with temperature and increased under higher temperature at the beginning. In addition, a proper temperature is beneficial for shortening the ripening time, producing the special aromatic flavor and improving the quality of the final products. For example, Pérez-Santaescolástica et al. [78] applied a mild thermal treatment (around $30{\;}^{\circ }C$) to avoid high instrumental adhesiveness and pastiness due to excessive proteolysis. Interestingly, as shown in Figure 1b,c, Zhao et al. [8] studied and found that there was an interactive effect between temperature and pH value. Specifically, the continuous decrease of water activity caused the decrease of protease activities, and the increment of maturation temperature increases the enzyme activities within a certain range. In the production, the management of drying rate is accomplished by relative humidity and the temperature of drying air. As mentioned in Table 2, the temperature is kept low until the water activity fall down to a level that suppresses microbial growth, which suggests a relatively low enzyme activity. During the period of drying, the temperature is increased to allow the increment in the proteases activities. It is an important stage for endogenous proteases to kick in.

### 4.3. Sodium Chloride Content and Processing Time

Sodium chloride plays an important role in regulating endogenous proteolysis. It is an indispensable material for salting processing, which functions mainly in the ways of seasoning, antiseptic and water retention improvement. However, excessive sodium intake will lead to high blood pressure and increase the incidence of heart disease and stroke [80]. The proper NaCl concentration is beneficial to the shelf life, safety characteristics, and the eating quality of cured meat products. However, lower cathepsin activity and excessive saltness mainly result from high salt content in the finished products. The increase in salt content and the loss of moisture result in the increment of hardness and chewiness of ham [81]. Thence, the problem of reducing salt content without affecting the palatability and safety of meat products has always been a hot research topic. Toldrá et al. [42] indicated that sodium chloride was a strong inhibitor for protease activity. Zhao et al. [8] also demonstrated that cathepsin B was susceptible to salt content, and the inhibitory effect will be enhanced with an increase in temperature. Specifically, higher temperatures increase the inhibitory effect on enzyme activity. Interestingly, Zhou et al. [21] found that salting treatment accelerated the release of cathepsin B from lysosomes and demonstrated that cathepsin B was sensitive to NaCl.

When it comes to the processing time, the activity of endogenous proteases is dynamic with the extension of time. Zhang et al. [82] found that the temperature and humidity were directly related to the dehydration rate and processing time. Ding et al. [83] showed that muscle protein undergone strong hydrolysis and the content of free amino acid increased over time. Salazar et al. [84] also proved that the content of free amino acids was positively correlated with the processing time. Moreover, the content of non-protein nitrogen and peptides increased over time. As described above, the endogenous protease activity in the hams is directly affected by the water activity, pH and temperature, and they are time-varying. During the whole processing of the Jinhua ham, the muscle pH increase slightly with time, and the temperature fluctuates in the range of 0 ${}^{\circ }C$–37 ${}^{\circ }C$. A significant decrease in endogenous protease activity occurs after salting. An important reduction of moisture content is typically observed, and Pugliese et al. [85] suggested that the salt content gradually increased over time. High sodium chloride content inhibited the growth of spoilage bacteria and produces a negative impact on the activity of endogenous proteases. Some proteases are relatively stable during the post-ripening period and not completely inactivated, which is due to the rising temperature and the destruction of organelles to release lysosomal proteases. The treatment time affects the molecular weight of peptides produced in ham. Hams with a shorter processing time have a higher molecular weight, while the molecular weight of hams with a longer processing time is smaller. This is because that large molecular weight peptides are further degraded into small molecular peptides with time [86].

### 4.4. Ultrasound-Assisted Technique and High-Pressure Processing during the Manufacture

There are some non-destructive techniques that have been evaluated with regard to their implementation in the manufacturing of dry-cured ham, including irradiation, magnetic resonance imaging, infrared spectroscopy (IR), high pressure processing (HPP), and ultrasound (US). Kang et al. [87] found that the diffusion coefficient of NaCl or water increases significantly with the increase of ultrasonic intensity in the range of 2.39–20.96 $W/{\mathrm{cm}}^{2}$, which is shown in Figure 5b. Furthermore, Refs. [88,89] showed that the ultrasound treatment caused damage to lysosomal membrane, which promoted the release of cathepsins and further improved their activities (Figure 5c).

In addition to the application in salting, there have been many reports of potential techniques for correcting and improving the organoleptic and textural attributes of defective ham. Sensory defects of ham are highly related to protein degradation catalyzed by endogenous proteases, which are located in the muscle tissues. The application of high-pressure technology has a key impact on the organoleptic characteristics of final products, which is based on the fact that high pressure will promote the changes in protein unfolding, amino acids releasing and cross-link between proteins. Buckow et al. [90] found that the activity of cathepsin B and L increased gradually by increasing the pressure to 400Mpa, which was due to the release of enzymes from muscle cells by the high pressure. When the processing pressure was increased to 600Mpa, the hardness increased and the pastiness and adhesiveness decreased in the dry-cured ham [91]. Rivas-Cañedo et al. [92] observed a higher proteolysis under 600Mpa and $35{\;}^{\circ }C$, resulting in further accumulation of free amino acids. After that, they also detected that the activity of cathepsin D would increase in the temperature range (33 ${}^{\circ }C$–70 ${}^{\circ }C$), indicating that high pressure promoted the rupture of lysosomal membranes and accelerated the release of cathepsins. Zhou et al. [93] illustrated that ultrasound-assisted thermal treatment improved the activities of aminopeptidases, which further accelerated the degradation of polypeptides into free amino acids and then the taste attributes were enriched. It is worth noting that the appropriate operating conditions should be selected, or else the nutrients may be degraded and a peculiar smell generated, causing damage to the quality of the products [94]. For instance, Kang et al. [95] showed that inappropriate processing significantly increased the lipid and protein oxidation and led to high-level protein structure changes during salting.

## 5. Conclusions

The activity of muscle endogenous proteases can be detected in the whole process, and they play an important role in Jinhua ham flavor and texture. The regulation of enzyme activities can be achieved by changing the processing conditions. At the optimum temperature or pH, the protease is the most active. Salting and decreased water activity in muscle are associated with the decrease in enzymatic activity. In addition, the length of treatment time is positively correlated with the degree of proteolysis, that is, the accumulation of flavor substances. Some new and developing processing techniques accelerate the release of endogenous proteases from cell membranes, such as high-pressure technology and ultrasound. However, it is a challenge to decide on appropriate processing parameters. In the future, for Jinhua hams, the quantitative relationship needs to be further explored between key process parameters and endogenous proteases activities. Additionally, the interaction between various factors involved in ham manufacturing cannot be ignored, and a more accurate method for enzyme activity determination is needed.

## Figures and Tables

**Table 1:** Partial characteristics of the common lysosome cathepsins existing in animal muscles [24,27].

Name	EC Number	Classification	Molecular Size	Optimal pH	Isoelectric Point	Feature
Cathepsin B	3.4.22.1	Cysteine protease	25–30 KDa	5.5–6.5	4.5–5.5	Peptidyl dipeptidase activity.
Cathepsin L	3.4.22.15	Cysteine protease	24–28 KDa	5.5–6.5	5.0–6.3	Strong proteolytic activity; weaker activity on synthetic substrates.
Cathepsin D	3.4.23.5	Aspartic protease	30–45 KDa	3.0–5.0	6.8	One of the most abundant cathepsins in the lysosome.
Cathepsin H	3.4.22.16	Cysteine protease	23–28 KDa	6.5–6.8	6.0–7.1	Strong aminopeptidase activity.
Cathepsin S	3.4.22.27	Cysteine protease	24 KDa	6.0–6.5	6.3–7.0	Vigorous and stable in weak alkaline environment (pH 7.5).
Cathepsin E	3.4.23.34	Aspartic protease	42 KDa	3.0–3.5	4.1	Exist only in macrophages of skeletal muscle.

**Table 2:** The processing parameters of Jinhua ham with modern technology [49,79].

Stage	Weight Loss	Temperature	Humidity	Time
Raw ham	0%	0–4 ${}^{\circ }C$	80–90%	2 d
Salting		0–4 ${}^{\circ }C$	70–80%	15 d
Pickling	14–16%	0–4 ${}^{\circ }C$	75–85%	40 d
Washing and air drying		10–15 ${}^{\circ }C$	60–70%	7 d
Maturation−1		10–20 ${}^{\circ }C$	65–80%	30 d
Maturation−2	28–30%	18–30 ${}^{\circ }C$	55–65%	40 d
Maturation−3	35–36%	30–35 ${}^{\circ }C$	60–65%	30 d
Post-ripening	40–42%	20–25 ${}^{\circ }C$	60–80%	30 d

## Data Availability

The data presented in this study are available on request from the corresponding author.
